# Richmond Academy of Medicine and Surgery

**Published:** 1895-06

**Authors:** M. W. Peyser

**Affiliations:** Secretary; Richmond, Va.


### SOCIETY REPORTS.

### RICHMOND ACADEMY OF MEDICINE AND SURGERY.

RICHMOND, VA., April 9, 1895.

*The President, Dr. Wm. S. Gordon, in the chair.*

This meeting was almost entirely devoted to the discussion of malarial, typhoid, and typho-malarial fevers. These were fully dissected, all points being brought out; and the consensus of opinion being that the last named disease should receive a new appellation.

DR. HUGH M. TAYLOR presented an appendix, removed on this date, which illustrated the now well-known fact, that an insidious mildness or even a total absence of symptoms not infrequently accompanied serious morbid changes. He knew of no trouble that presented a greater number of surgical surprises.

The young man upon whom he had just operated had been the subject of three attacks of appendicitis during the past eighteen months, and had been attended in each by Dr. Miller, of Clifton Forge, Va. When admitted to Virginia Hospital, he was the picture of health; had absolutely no pain on deep pressure, no fever, or any other evidence of existing inflammation.

DR. TAYLOR said he had not often gone into a belly with more reluctance than in this case. He thought it required a keen appreciation of the dangers of conservatism to take a man in apparently perfect health and subject him to even the small risk of appendectomy in the quiescent stage. Any existing scruples must, however, vanish before the conviction that the disease is an exudative infection—a load of dynamite liable to explode at any moment with disastrous consequences; and so often the violence of the explosion is out of all proportion to the warnings manifested.

In the case reported, the appendix was found bound to a considerable extent by adhesions, and in its entire length was enlarged to the size of the index finger. Its lumen was filled with mucopurulent fluid; its walls very much thinned, and at one point necrosed to such an extent that it needed but a very little handling to cause a rupture.

The interest in the case centers in the fact that we can have so serious a destructive change with so few indications, and confirms the statement so pointedly brought out by Treves in a recent address, that the severity and number of attacks of appendicitis is no gauge to the extent of adhesions, etc., likely to be encountered. In not a few instances after one attack, the appendix has been found buried in a mass of adhesions. On the other hand, it may be found comparatively free after repeated inflammations.

The dangers of appendectomy during the quiescent stage pale into insignificance when contrasted with those incident to diffuse, septic or suppurative peritonitis; the more frequently we encounter the latter, the less we dread the former.

MEETING APRIL 23, 1895.

*The President, Dr. Wm. S. Gordon, in the chair.*

DR. J. ALLISON HODGES, leader in the subject selected for the evening's discussion, read a paper on some of the diagnostic nervous manifestations of syphilis.

The nervous symptoms are more manifest in proportion to the absence of cutaneous symptoms. All the nervous symptoms are not always dependent on syphilis; a number of nervous diseases have their origin in this malady. In diagnosing the disease, the medication method is unreliable, some other affections being improved by it. The nervous symptoms may be developed in each stage of the disease, but it is in the tertiary stage principally that the gravest lesions of the nervous system appear; and, since it is especially in those cases where the ordinary secondary manifestations were wanting that we are to expect these complications, it is important for physicians, in making such a diagnosis, to be prepared to recognize the first danger signals that may be manifested. The primary stage has no prominent nervous symptoms, those present being referable rather to the concomitant anemia than to the action of the specific poison. The secondary stage presents more marked evidences of implication of the nervous system; various neuralgias, dyspepsia of nervous origin, cardiac palpitations, and meningitis, cerebral or spinal, being characteristically present. The tertiary stage gives evidence of numberless shades and varieties of nervous affection; and in this period of the disease the nervous symptoms manifested are due solely to the influence of the specific virus circulating in the blood and irritating the delicate nervous structures.

The symptoms produced may be those due to an inflammation or degeneration of the nerve centers themselves, or to the effects produced by pressure upon the nerve centers, or trunks, by product of this same form of inflammation located in contiguous structures; the symptoms all showing lesions either of the intra-cranial organs or of the spinal cord, less frequently of the spinal nerves.

The diagnostic symptoms detailed, are also diagnostic of other diseases. It is by association that we determine the disease, as locality, etc. There are periodic occipital headaches in nearly every case; absent in the forenoon, returning most frequently at night and becoming worse. I have never found any tenderness on pressure. The diagnostic nervous manifestations of syphilis are: 1. Headache, which disappears if paralysis occurs. 2. Insomnia, nearly always associated with headache, and disappearing with the appearance of convulsions or paralysis. It differs from the insomnia of neurasthenia and melancholia in that it occurs in the early night, the victim arising in the morning ready for his daily labor. 3. Vertigo, occurring usually with the headache. It may be transient, but becomes worse as the disease progresses. 4. Convulsions. In the adult they are not preceded by convulsions in youth. 5. Tremor, present in one-half of cases. It occurs most often, in the order named, in the hands, tongue, and over the whole body, and is accompanied by headache. If it occurs in a limb, it is the precursor of paralysis of the limb. 6. Hemiplegia. 7. Erratic distribution of paralysis, as aphasia with or without hemiplegia; ptosis; insanity or epilepsy with paralysis of one arm or lèg. It is suggested that ptosis occurring suddenly points nearly always to syphilis. 8. The use of electricity to determine central or peripheral lesion. 9. The presence of great physical weakness and mental dullness. This is one of the most valuable of the nervous manifestations, being out of proportion to the seeming condition of the patient. 10. History of the case. In women the history of many abortions in succession would point to syphilis.

In the treatment of syphilis, the iodide should be given in sufficiently potential doses in Carlsbad or other waters.

In conclusion, let me say, we could often abort syphilis by studying the nervous system and giving treatment in time.

M. W. PEYSER, M.D., Secretary.

